# Positron emission tomography to assess drug occupancy at peripheral and central incretin receptors

**DOI:** 10.1016/j.ebiom.2025.106033

**Published:** 2025-11-21

**Authors:** Amina Khalil, Irina Velikyan, Mengfei Xiong, Martin Bossart, Michael Wagner, Olof Eriksson

**Affiliations:** a Science for Life Laboratory, Department of Medicinal Chemistry, Uppsala University, Uppsala, Sweden; b PET-Centre, Centre for Medical Imaging, Akademiska Sjukhuset, Uppsala, Sweden; c Department of Surgical Sciences, Radiology and Molecular Imaging, Uppsala University, Uppsala, Sweden; d Integrated Drug Design, Synthetic Medicinal Modalities, Sanofi, Frankfurt, Germany; e Dewpoint Therapeutics, Frankfurt, Germany; f Antaros Tracer AB, Uppsala, Sweden

**Keywords:** GLP-1R, Exendin-4, Tirzepatide, SAR441255, Incretins

## Abstract

**Background:**

Incretin mimetics, especially dual/triple agonists, are effective for type 2 diabetes and obesity, though mechanisms remain unclear. This study applied PET using [^68^Ga]Ga-DO3A-Exendin-4 and [^68^Ga]S02-GIP-T4 to assess GLP-1R and GIPR occupancy of SAR441255 (a GLP-1R, GIPR, and GCGR agonist) and tirzepatide in pig pancreas and CNS.

**Methods:**

*In vitro* binding assays on frozen HEK293 cell sections overexpressing human GLP-1R, GIPR, or GCGR assessed [^68^Ga]Ga-DO3A-Exendin-4 specificity and competition with tirzepatide and SAR441255, and [^68^Ga]S02-GIP-T4 with SAR441255. *In vivo*, the GLP-1R occupancy by SAR441255 and tirzepatide, and GIPR occupancy by SAR441255, was evaluated in healthy pigs using PET/CT initiated at tracer injection. A subcutaneous dose of the study drug was then administered, and a second scan was performed 2.5 h later. Occupancy was determined by comparing pancreatic and CNS tracer SUV before and after dosing. Two animals were used to compare the tracers directly.

**Findings:**

[^68^Ga]Ga-DO3A-Exendin-4 and [^68^Ga]S02-GIP-T4 showed high specificity for GLP-1R and GIPR, respectively, and competed with the study drugs *in vitro*. *In vivo*, SAR441255 induced dose-dependent GLP-1R occupancy (>70%) in pancreas and pituitary and (>60%) in CNS, while tirzepatide showed lower occupancy. SAR441255 also reduced pancreatic [^68^Ga]S02-GIP-T4 uptake by 23 ± 8.5%, indicating GIPR engagement.

**Interpretation:**

PET imaging in pigs demonstrated *in vivo* GLP-1R engagement by SAR441255 and tirzepatide, and GIPR engagement by SAR441255 in the pancreas. SAR441255 exhibited dose-dependent GLP-1R occupancy in the pancreas and brain regions linked to appetite regulation.

**Funding:**

The study was funded by Uppsala Diabetes Center, 10.13039/501100008550Diabetesfonden, ExoDiab, Diabetes Wellness Sweden, 10.13039/501100004973Barndiabetesfonden, 10.13039/501100009252Science for Life Laboratory, and the 10.13039/501100004359Swedish Research Council.

**Research in context**

**Evidence before this study**

Multi-incretin receptor agonists are a new class of therapeutic agents that have demonstrated promising results in improving glycaemic control, promoting significant weight loss, and regulating appetite in individuals with type 2 diabetes and obesity. Recent studies suggest that these agents act not only on peripheral incretin receptors but also within the central nervous system. However, the precise mechanisms underlying their central effects, including dose-dependent receptor occupancy and signalling pathways, remain unclear.

**Added value of this study**

This study demonstrates the ability to non-invasively monitor both peripheral and central, dose-dependent GLP-1R occupancy by dual and triple incretin receptor agonists *in vivo* using a GLP-1R–specific PET radiotracer. Additionally, it enables the evaluation of peripheral GIPR occupancy by a triple incretin agonist through the application of a GIPR-specific radiotracer. Furthermore, the study investigates the suitability of an animal model for conducting translational receptor occupancy studies involving incretin-based therapies.

**Implications of all the available evidence**

The findings from this study demonstrate the ability to monitor dose-dependent *in vivo* target engagement of both dual and triple incretin receptor agonists at the GLP-1R, as well as engagement of dual agonists at the GIPR. The observed dose-dependent GLP-1R occupancy in both peripheral tissues and central appetite-regulating regions of the brain provides important insight into the action of multi-incretin mimetic drugs. Our data further highlight the suitability of a porcine model for GLP-1R occupancy studies, although its relatively low pancreatic GIPR expression may limit its utility for evaluating GIPR engagement. Overall, this study provides a robust imaging approach that is directly translatable to humans, offering a valuable tool for assessing receptor engagement and guiding dose optimisation during the clinical development of incretin-based therapies.

## Introduction

Incretin mimetics are a class of medications that mimic the action of physiological incretin hormones, i.e., glucagon-like peptide-1 (GLP-1) and glucose-dependent insulinotropic polypeptide (GIP).^1^ The incretin hormones are produced in the gastrointestinal tract and released into the bloodstream in response to oral food intake.^2^ The presence of the GLP-1 receptor (GLP-1R) and GIP receptor (GIPR) was initially demonstrated in the pancreatic beta cells, where activating these receptors augmented insulin production.^3^

However, GLP-1R and GIPR are distributed throughout the body at various levels in different organs and tissues such as adipose tissues, heart, kidneys, lungs, intestines, and the central nervous system (CNS).^4^ In the CNS, GLP-1R is mainly found in tissues without blood brain barrier (BBB), such as the circumventricular organs (CVOs), or brain regions only partially protected by the BBB, including the arcuate nucleus and paraventricular nucleus in the hypothalamus (e.g., the “appetite centre” in the brain).^5^ In addition to their insulinotropic effect, incretin mimetics have demonstrated positive effects on pancreatic β-cell function and proliferation, decreased body weight, plasma lipid profile, and blood pressure.^6^ They have also shown the potential to delay the risk of developing cardiovascular diseases.^7^

Incretin mimetics are usually recommended when the glucose-lowering effect of an oral medication alone is not sufficient.^8^ They might also be prescribed to avoid hypoglycaemia and weight gain that can be associated with some oral diabetic medications, as well as for patients with a high risk of atherosclerotic cardiovascular disease.^8^

The success and favourable safety profile of mono GLP-1R agonists such as liraglutide and semaglutide have propelled the development of the next generation of incretin mimetics, such as unimolecular dual and triple receptor agonists that target a combination of the GLP-1R, GIPR, and/or the glucagon receptor (GCGR). However, while mono agonists have proven effective, their efficacy is limited by targeting a single metabolic pathway. In contrast, dual and triple receptor agonists can exert their effects through multiple pathways at the same time, leading to stronger and broader effects on blood sugar control, appetite, and weight management.^9^^,^^10^

Tirzepatide is the first dual GLP-1R/GIPR agonist approved for the treatment of type 2 diabetes (T2D).^11^ Tirzepatide demonstrated a greater glucose-lowering effect as well as greater weight reduction compared to mono GLP-1R agonism.^12^

Another multi-incretin receptor agonist, SAR441255, was designed to simultaneously activate GLP-1R, GIPR, and GCGR.^13^ This drug design approach allows for maximising the insulinotropic effect by activating both GLP-1R and GIPR while enhancing the metabolic balance, energy expenditure, and appetite regulation by including GCGR agonism.^14^

Even though the concept of incretin mimetic drugs is not new, their mechanism of action is still unclear, especially for dual and triple agonists. Agonism at the GLP-1R, GIPR, and GCGR has overlapping pharmacology, with each receptor contributing to glucose control and weight reduction.^15^ It is therefore challenging to pinpoint effects on established clinical endpoints to drug occupancy at a certain receptor. To better understand the binding properties of incretin mimetics, Positron emission tomography (PET), often combined with the computer tomography (CT), can be used as a tool to quantify and visualise the binding of the drug to its receptors, as well as show the distribution of the receptors in the body.^16^^,^^17^ PET can also provide data on pharmacokinetic parameters such as drug biodistribution in the organs and clearance pathways.^18^

This study aimed to evaluate the target engagement and dose-dependent occupancy of tirzepatide and SAR441255 at the GLP-1R in both the pancreas and CNS, as well as the target engagement and occupancy of SAR441255 at a high dose in the same tissues. A large animal pig model was selected for this study due to its high biological similarity to humans. However, it is important to note that GIPR expression in the pancreas of pigs is lower compared to humans (Supplementary Figure S1), which may represent a limiting factor in evaluating pancreatic GIPR occupancy of SAR441255 in this model.

The study was performed by using [^68^Ga]Ga-DO3A-Exendin-4, a highly specific GLP-1R agonist, as a radiotracer for PET imaging to track the binding affinity of these drugs to GLP-1R.^19^^,^^20^ To assess occupancy at the GIPR, [^68^Ga]-DOTA-S02-GIP-T4, a newly developed PET radioligand, was used. Previous studies have demonstrated that [^68^Ga]S02-GIP-T4 exhibits high affinity for GIPR both *in vitro* and *in vivo* in non-human primate models.^16^

## Methods

### Radiochemistry

The radiolabelling synthesis and quality control of [^68^Ga]Ga-DO3A-Exendin-4 was conducted according to the method published earlier.^21^ Similarly, the radiosynthesis of [^68^Ga]S02-GIP-T4 was performed according to a previously published method.^22^

### *In vitro* autoradiography specificity assay

#### GLP-1R tracer [^68^Ga]Ga-DO3A-Exendin-4

An *in vitro* binding assay was performed to verify firstly that [^68^Ga]Ga-DO3A-Exendin-4 is specific for the GLP-1R, and secondly to demonstrate that tirzepatide and SAR441255 compete with the tracer for binding to GLP-1R. Both these features are required to use [^68^Ga]Ga-DO3A-Exendin-4 for *in vivo* PET studies of GLP-1R target engagement.

To confirm the binding specificity of [^68^Ga]Ga-DO3A-Exendin-4 to GLP-1R, a blocking assay was conducted using sections of frozen pellets of HEK293 cells (RRID:CVCL_0045) overexpressing either human GLP-1R (huGLP-1R-HEK293), GIPR (huGIPR-HEK293) or GCGR (huGCGR-HEK293) (Evotec AG, Hamburg, Germany). The sections were incubated with 10 nM [^68^Ga]Ga-DO3A-Exendin-4 either alone or pre-incubated (10 min in PBS + 1% BSA), with 1 μM of DO3A-Exendin-4 (generated by custom in-house Solid Phase Peptide Synthesis (SPPS) by Sanofi), for blocking, in 150 ml of PBS + 1% Bovine Serum Albumin (BSA) for 60 min at room temperature (RT). The receptor specificity assay was repeated four times.

To assess if tirzepatide and SAR441255 compete with [^68^Ga]Ga-DO3A-Exendin-4 for binding to GLP-1R, frozen huGLP-1R-HEK293 cell pellet sections were pre-incubated with either 10 μM tirzepatide, 10 μM SAR441255 (both compounds were generated by custom in-house Solid Phase Peptide Synthesis (SPPS) by Sanofi), or 1 μM DO3A-Exendin-4 (positive control). Following pre-incubation, 10 nM of [^68^Ga]Ga-DO3A-Exendin-4 was added to the incubation buffer, and the sections were allowed to incubate for 60 min at room temperature (RT).

The following procedures were then performed for both assays: After incubation, the sections were washed with cold PBS (3× 1 min), dipped in MilliQ water, and air-dried at 37 °C for 10 min. Finally, the sections were exposed to digital phosphorimager plates (BAS-MS, Fujifilm) overnight (>10 radioactive half-lives) followed by digitalisation using a phosphorimage scanner (Amersham Typhoon FLA 9500 Phosphor Imager, GE) with a 50-μm pixel size for image visualisation. A 10 μl reference droplet, containing a known amount of radioactivity, was incorporated to facilitate the quantification of the autoradiogram.

The acquired images were analysed using ImageJ software (version 1.53k). The pixel values, measured in counts/mm^2^, were transformed into Bq/mm^2^ using the internal reference. Subsequently, the Bq/mm^2^ values were further converted to fmol/mm^2^ based on the molar activity (Bq/fmol) of each [^68^Ga]Ga-DO3A-Exendin-4 synthesis. The total binding to each cell section was compared to the residual binding remaining in cell sections treated with either DO3A-Exendin-4, tirzepatide or SAR441255, to measure the percentage of blocked tracer binding.

#### GIPR tracer [^68^Ga]S02-GIP-T4

*In vitro* autoradiography using sections of frozen pellets of huGIPR-HEK293 overexpressing GIP receptors was similarly performed to confirm that [^68^Ga]S02-GIP-T4 and SAR441255 compete for the same binding site on GIPR. Following the experimental approach described in the previous section, the sections were incubated for 60 min at RT either with 10 nmol of [^68^Ga]S02-GIP-T4 tracer alone, or in the presence of 10 μM of SAR441255 or 10 μM of unlabelled DOTA-S02-GIP-T4 (generated by custom in-house Solid Phase Peptide Synthesis (SPPS) by Sanofi), for positive control, to pre-block the binding sites. After the incubation, the sections were washed, exposed to digital phosphorimager plates, and analysed as described in the previous section.

### Ethics

All the animal experimentations and handling were approved by the regional animal ethical committee and performed according to the Uppsala University guidelines for animal research (UFV 2007/724) and in respect of the ARRIVE guidelines.

### Animal handling and anaesthesia

Animal experiments in this study were carried out in healthy pigs.

The pigs (n = 16 in total, Yorkshire × Swedish Landrace × Hampshire, weight 30.7 ± 2 kg) were transported from the farm to the PET facilities on the morning of the experiment and anaesthetised by intravenously injecting a combination of fentanyl, ketamine, and midazolam. An endotracheal tube was placed into the trachea to assist ventilation. An arterial catheter was placed for blood sampling (1 ml) to measure tracer activity in whole blood and plasma at 5, 30, 60, and 90 min post-tracer injection. Several venous catheters were placed for PET tracer injection and infusion of anaesthesia.

For each experimental condition, one pig was used, except for the [^68^Ga]Ga-DO3A-Exendin-4 and [^68^Ga]S02-GIP-T4 comparison (n = 2) and the GIPR drug occupancy study using [^68^Ga]Ga-S02-GIP-T4 (n = 5), where additional animals were included to evaluate reproducibility. Sample sizes were selected to minimise animal use in accordance with the 3Rs principle while ensuring sufficient data to meet the primary study objectives.

### Comparative biodistribution of [^68^Ga]Ga-DO3A-Exendin-4 and [^68^Ga]S02-GIP-T4 in pigs

In order to validate the use of the two tracers in pigs, before initiating drug occupancy studies, a direct comparison of their biodistribution and *in vivo* binding in relevant tissues was performed in pigs (n = 2).

Each pig received both [^68^Ga]Ga-DO3A-Exendin-4 and [^68^Ga]S02-GIP-T4 on the same day. Each pig was first injected with the GIPR-specific radiotracer, [^68^Ga]S02-GIP-T4, in the morning, followed by an injection of the GLP-1R-specific radiotracer, [^68^Ga]Ga-DO3A-Exendin-4, in the afternoon, with a minimum interval of 4 h between the injections to allow for excretion and decay of the first tracer administration. This approach allowed a direct *in vivo* comparison of the two tracers, enabling differentiation of the distribution of GIPR and GLP-1R (Table 1). This experimental design enabled the selection of optimal imaging time points for each PET tracer across different tissues.

**Table 1:** Experimental conditions for [^68^Ga]S02-GIP-T4 vs [^68^Ga]Ga-DO3A-Exendin-4 comparison in pigs (n = 2).

Animal #	Weight (kg)	Scan 1		Scan 2	
		[^68^Ga]S02-GIP-T4		[^68^Ga]Ga-DO3A-Exendin-4	
		(MBq/kg)	(MBq/nmol)	(MBq/kg)	(MBq/nmol)
1	29.9	0.6	28.6	0.8	34.3
2	34.6	1	46.8	1	48.1

### *In vivo* GLP-1R drug occupancy in pigs using [^68^Ga]Ga-DO3A-Exendin-4

#### Baseline scan

Pigs (n = 9) were positioned in the PET/CT scanner (Discovery MI, GE Healthcare) in a supine position with the brain at the centre of the Field of View (FOV). An attenuation CT scan of the brain and pancreas was performed, followed by an intravenous injection of 0.5–1 MBq/kg [^68^Ga]Ga-DO3A-Exendin-4 (corresponding to a standardised target dose 0.1 μg/kg DO3A-Exendin-4 peptide mass). The peptide mass dose was chosen to avoid self-block of the GLP-1R by unlabelled DO3A-Exendin-4 peptide in the formulation and was based on previous dose-escalation studies in non-human primates.^23^ A dynamic 60-min PET scan was started at tracer injection, serving as the baseline scan to visualise and quantify GLP-1R expression under normal physiological conditions prior to drug intervention. Following the dynamic scan, a whole-body CT was acquired, immediately followed by a whole-body static PET scan covering the regions from the brain to the bladder. For experimental details and injected amounts of radioactivity in each experiment, see Table 2, Table 3, Table 4.

**Table 2:** Experimental conditions for the pigs (n = 5) treated with SAR441255, imaged using [^68^Ga] Ga-DO3A-Exendin-4 tracer.

Animal #	Weight (kg)	Scan 1		Scan 2		SAR441255 (μg/kg)
		[^68^Ga]Ga-DO3A-Exendin-4		[^68^Ga]Ga-DO3A-Exendin-4		
		(MBq/kg)	(MBq/nmol)	(MBq/kg)	(MBq/nmol)	
4	30.7	1	44.1	1.2	51.9	1
5	26.4	0.6	24.9	0.9	42.1	2.5
6	33	0.9	40.4	1.2	54.5	4
7	28.9	0.8	35.5	0.9	40.3	14
8	33.5	1.2	52.8	0.9	40.3	100

**Table 3:** Experimental conditions for the pigs (n = 3) treated with tirzepatide, imaged using [^68^Ga] Ga-DO3A-Exendin-4 tracer.

Animal #	Weight (kg)	Scan 1		Scan 2		Tirzepatide (μg/kg)
		[^68^Ga]Ga-DO3A-Exendin-4		[^68^Ga]Ga-DO3A-Exendin-4		
		(MBq/kg)	(MBq/nmol)	(MBq/kg)	(MBq/nmol)	
9	30.5	0.8	35.3	1	45.0	50
10	30	0.9	39.6	1.1	51.6	150
11	30.6	1	43.8	1.1	51.0	450

**Table 4:** Experimental conditions for the pig (n = 1) injected with unlabelled DO3A-Exendin-4 for self-blocking, as a positive control, imaged using [^68^Ga]Ga-DO3A-Exendin-4 tracer.

Animal #	Weight (kg)	Scan 1		Scan 2		DO3A-Exendin-4 (μg/kg)
		[^68^Ga]Ga-DO3A-Exendin-4		[^68^Ga]Ga-DO3A-Exendin-4		
		(MBq/kg)	(MBq/nmol)	(MBq/kg)	(MBq/nmol)	
3	34.7	0.7	33.1	1.3	57.1	10

#### Study drug administration

After completion of the baseline PET scan (≈2 h after the baseline scan tracer injection), each pig received a subcutaneous injection of a study drug: either 1–100 μg/kg of SAR441255 (Table 2) or 50–450 μg/kg of tirzepatide (Table 3). One experiment used unlabelled DO3A-VS-Exendin-4 (6 μg/kg) as a positive control (Table 4).

At least 2.5 h after the study drug administration, a second dynamic PET scan was performed. A waiting period of 2.5 h was selected for two primary reasons. First, this duration allows the study drugs to distribute and bind to the target receptors, aligning with the reported time to maximum concentration (T_max_) for SAR441255, which occurs within a few hours after administration.^13^ In contrast, tirzepatide exhibits a significantly longer T_max_, on the order of days, due to its extended plasma half-life, and is therefore not expected to reach its maximum concentration (C_max_) within this timeframe. To achieve exposures comparable to those expected in humans, higher doses of tirzepatide, up to 450 μg/kg, were administered to compensate for the prolonged T_max_.

Second, the 2.5 h waiting period allowed sufficient time from the baseline tracer injection for the excretion and radioactive decay of the first injection of [^68^Ga]Ga-DO3A-Exendin-4.

By this time, at least 4 h (approximately 4 radioactive half-lives for Gallium-68, t_1/2_ = 68 min) had passed from the first tracer injection, and thus less than 6% of the radioactivity remains, excluding excretion. Thus, negligible amounts of the tracer remained in the body at this time, ensuring no significant interference with the follow-up (on-drug) scan.

#### Follow-up scan

The on-drug follow-up scan was performed identically as the baseline scan by administration of a second injection of 0.5–1 MBq/kg of [^68^Ga]Ga-DO3A-Exendin-4 (target peptide dose of 0.1 μg/kg) (Table 2, Table 3, Table 4). The follow-up scan was designed to assess the decreased binding of the tracer to its receptor, which, when compared to the baseline scan, would indicate reduced receptor availability, such as that caused by drug occupancy at the target GLP-1 receptor.

After the second follow-up PET scan was completed, a contrast-enhanced CT scan over the pancreas and brain was performed at the arterial and venous phases for optimal anatomical images of soft tissues to assist with tissue delineation.

### In vivo GIP drug occupancy in pigs using [^68^Ga]Ga-DOTA-S02-GIP-T4

#### Baseline scan

The study design for GIPR occupancy assessment in pigs was identical to that of GLP-1R, except for the following differences:

The pigs (n = 5) were positioned in the PET/CT scanner in a supine position with either the brain (n = 2) or the pancreas (n = 3) in the FOV when using [^68^Ga]S02-GIP-T4. A target dose of 0.5–1 MBq/kg of [^68^Ga]S02-GIP-T4, corresponding to a standardised peptide mass dose of 0.1 μg/kg, was administered for both baseline and follow-up scans (Tables 5 and 6). The peptide mass dose was chosen to avoid self-block of the GIPR, and based on previous dose-escalation studies in non-human primates.^16^

**Table 5:** Experimental conditions for pigs (n = 4) treated with SAR441255 and imaged using [^68^Ga]S02-GIP-T4 tracer.

Animal #	Weight (kg)	Scan 1		Scan 2		SAR441255 (μg/kg)
		[^68^Ga]S02-GIP-T4		[^68^Ga]S02-GIP-T4		
		(MBq/kg)	(MBq/nmol)	(MBq/kg)	(MBq/nmol)	
13	31	0.8	36.7	1.1	51.4	100
14	30	1	43.5	1	46.5	100
15	31.9	0.6	28.5	0.6	28.4	100
16	26.6	0.7	32.0	0.9	42.5	100

**Table 6:** Experimental conditions for pig (n = 1) treated with a 30-min infusion of endogenous GIP (1–42), imaged with [^68^Ga]S02-GIP-T4 tracer.

Animal #	Weight (kg)	Scan 1		Scan 2		GIP (1–42) 30-min infusion (μg/ml)
		[^68^Ga]S02-GIP-T4		[^68^Ga]S02-GIP-T4		
		(MBq/kg)	(MBq/nmol)	(MBq/kg)	(MBq/nmol)	
12	28.6	0.7	29.6	0.8	34.1	0.5/50

#### Follow-up scan

SAR441255 was administered subcutaneously at a dose of 100 μg/kg in four pigs, as described above (Table 5). The high dose was chosen to reach maximal occupancy. One pig out of 5 was used in a positive control study to validate the [^68^Ga]S02-GIP-T4 tracer in pigs, where the animal received an infusion of GIP (1–42) (Sigma-Aldrich, Cat. No. G2269) (0.5/50 μg/ml), an endogenous biological GIPR ligand, starting 30 min before and continuing throughout the second scan (Table 6).

### Detailed imaging acquisition and reconstruction protocol

For the high dose CT scans of the brain, the pancreas or whole body, the following parameters were used: 100 kV, 80–400 mA, noise index 10, rotation 0.5′, full spiral, slice thickness 3.75 mm, pitch 0.98:1, recon diameter 30 cm.

The PET acquisition and reconstruction protocol for the dynamic scans over pancreas and brain was as follows: 25 cm axial FOV, 60-min scan in list mode, 30 frames of 12 × 10′, 6 × 30′, 5 × 2′, 5 × 5′, 2 × 10′, VPFX-S, 3 i/16 s, 256 × 256 pixels, 3 mm post filter, 30 cm diameter zoom.

The PET whole body scan used the following reconstruction parameters: VPFX-S, 3 i/16 s, 256 × 256 pixels, 3 mm post filter, 30 cm diameter zoom.

### PET data analysis

The reconstructed dynamic and whole-body static PET images were analysed using PMOD Base Functionality (PBAS) tool (PMOD Technologies LLC, Zurich, Switzerland) through manual segmentation on trans-axial PET and CT projections. Segmented tissues included the descending aorta, pancreas, liver, spleen, kidneys, and muscle. The tracer distribution in brain regions was measured by automated segmentation of brain regions. This was achieved by fusing the acquired PET dynamic images with the PMOD MRI pig brain atlas. Due to the smaller size of the pig brain, combined with the limited resolution of clinical PET scanners (3–4 mm), very small brain regions cannot be distinguished in the PET images. As a result, regions like the ‘Medial hypothalamic area’ appear as aggregates in the PMOD pig brain atlas when applied to PET data. This area includes several anatomically distinct regions, such as the arcuate nucleus and the periventricular nucleus, both being part of the appetite centre of the brain, with known expression of GLP-1R. CVOs, such as pituitary gland, were not included in the brain atlas and were thus segmented manually.

The [^68^Ga]Ga-DO3A-Exendin-4 and [^68^Ga]S02-GIP-T4 uptake in each tissue for each scan was quantified in becquerels per cubic centimetre (Bq/cc) and converted to standardised uptake values (SUV). The convention to the SUV was corrected to the time and administered amount of radioactivity (in MBq) and the weight (in kilograms) of the pig.

The target engagement or drug occupancy for each experiment was calculated by subtracting the SUV in a tissue at the follow-up scan from the SUV in the same tissue at the baseline scan. The difference was divided by the SUV in tissue at baseline and multiplied by 100 to reach the percentage of drug occupancy (Equation 1):

Equation 1 $$Occupancy\;\left(\%\right)=\frac{{SUV}_{baseline}-{SUV}_{follow-up}}{{SUV}_{baseline}}\;\times \;100$$

The plasma-to-whole blood ratio for both [^68^Ga]Ga-DO3A-Exendin-4 and [^68^Ga]S02-GIP-T4 was >1 throughout the scan duration, indicating that a greater fraction of both tracers remains in the plasma, thereby increasing their availability for tissue uptake.

### Statistics

All PET measurements were expressed as average SUV_mean_. Statistical analyses were conducted using GraphPad Prism 10. For *in vitro* experiments, grouped differences were evaluated with an unpaired t-test using Ordinary one-way ANOVA. For *in vivo* tracer comparison experiments in individual tissues, uptake differences between the two tracers were assessed using a paired t-test, n = 2. A *p*-value < 0.05 was considered statistically significant. Significance levels are indicated as follows: *p* < 0.05 (∗), *p* < 0.01 (∗∗), *p* < 0.001 (∗∗∗), and *p* < 0.0001 (∗∗∗∗). Normality of distribution for *in vitro* data was assessed using the Shapiro–Wilk test, where a *p*-value >0.05 indicated normal data distribution.

For receptor occupancy experiments, nonlinear regression was used to fit a four-parameter logistic curve (EC_50_ model) relating drug dose to occupancy. Raw dose values were used, and the Bottom parameter was fixed at 0% occupancy to reflect biological assumptions, while Top, EC_50_, and Hill slope were freely estimated. Goodness of fit was assessed using R^2^ values.

This model was chosen due to its widespread use in pharmacology and receptor-binding studies, where it effectively captures the sigmoidal dose–response relationship and allows reliable estimation of EC_50_ and Hill slope.^24^^,^^25^

### Role of funders

The funders had no role in study design, data collection, data analysis, interpretation of data, or writing of the report.

## Results

### *In vitro* autoradiography specificity assay

#### Binding to GLP-1R

**Fig. 1:**
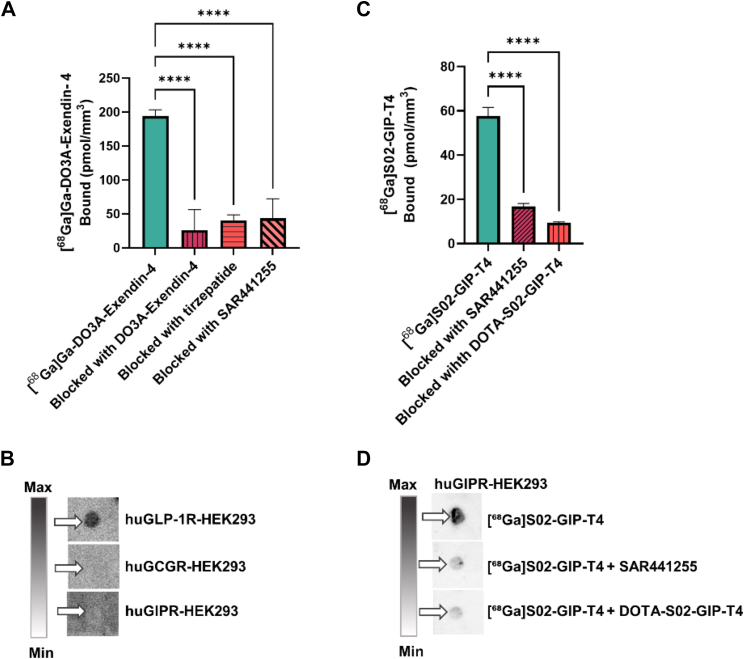
A) The uptake of [^68^Ga]Ga-DO3A-Exendin-4 in huGLP-1R-HEK293 alone or when co-incubated with either DO3A-Exendin-4, tirzepatide, or SAR441255 (∗∗∗∗*p* < 0.0001, R^2^ = 0.94, by one-way ANOVA). (B) Autoradiograms displaying [^68^Ga]Ga-DO3A-Exendin-4 uptake in frozen sections of huGLP-1R-HEK293, huGIP-R-HEK293, and huGCG-R-HEK293. (C) Binding of [^68^Ga]S02-GIP-T4 to huGIPR-HEK293 cell pellets, either incubated alone or in the presence of SAR441255 or DOTA-S02-GIP-T4 (∗∗∗∗*p* < 0.0001, R^2^ = 0.99, by one-way ANOVA). (D) Autoradiograms depicting the uptake of [^68^Ga]S02-GIP-T4 in frozen sections of huGIPR-HEK293 cells under different conditions. Data were normally distributed as assessed by the Shapiro–Wilk test (*p* > 0.05).

[^68^Ga]Ga-DO3A-Exendin-4 demonstrated strong binding to huGLP-1R-HEK293, with negligible cross-reactivity for huGIPR-HEK293 or huGCGR-HEK293 cell pellets, confirming previous *in vitro* findings of selective GLP-1R binding in human pancreas.^20^ The binding to huGLP-1R-HEK293 frozen pellets was reduced by 80 ± 16% (*p* < 0.0001, one-way ANOVA) when pre-incubated with 1 μM of DO3A-Exendin-4 (Fig. 1A and B). Pre-incubation with 10 μM tirzepatide or 10 μM SAR441255 reduced the binding of [^68^Ga]Ga-DO3A-Exendin-4 to huGLP-1R-HEK293 frozen pellets by 84.9 ± 7% and 78.3 ± 11.1%, respectively (*p* < 0.0001, one-way ANOVA) (Fig. 1A and B), demonstrating that each of the drugs and [^68^Ga]Ga-DO3A-Exendin-4 competed for an overlapping binding site of GLP-1R.

#### Binding to GIPR

[^68^Ga]S02-GIP-T4 exhibited strong binding to the huGIPR-HEK293 cell pellets. The binding of the tracer to the cell pellets was reduced by 71.2 ± 1% (*p* < 0.0001, one-way ANOVA) when pre-incubated with 10 μM SAR441255 and by 82.5 ± 0.9% (*p* < 0.0001, one-way ANOVA) when pre-incubated with 10 μM DOTA-S02-T4 (Fig. 1C and D). In the previously conducted study,^16^ [^68^Ga]S02-GIP-T4 demonstrated specific binding to huGIPR, with negligible uptake in HEK293 cells transfected with huGLP-1R or huGCG.

### PET assessment of i*n vivo* drug occupancy

#### Occupancy at the GLP-1R

The GLP-1R occupancy for SAR441255 and tirzepatide at various administered doses, as well as the occupancy of DO3A-VS-Exendin-4, was quantitatively assessed by calculating the reduction in [^68^Ga]Ga-DO3A-Exendin-4 uptake post-administration of the respective study drugs compared to the tracer's baseline uptake.

A detailed time activity data for each drug and corresponding radio-tracer is presented in Appendix A, Appendix A, Appendix A, Appendix A, Appendix A.

##### DO3A-VS-Exendin-4 control

Pre-treatment with unlabelled DO3A-VS-Exendin-4 served as a positive control to confirm the GLP-1R-specific binding of [^68^Ga]Ga-DO3A-Exendin-4 in tissues and organs known to express GLP-1R. Following pre-administration of DO3A-VS-Exendin-4, the uptake of [^68^Ga]Ga-DO3A-Exendin-4 decreased by 73% in the pancreas and by 53% in the pituitary gland (Supplementary Figure S2). In the circumventricular organs (CVO), tracer uptake was reduced by 55% in the hypothalamic region and markedly by 60% in the mammillary body (Supplementary Figure S2).

**Fig. 2:**
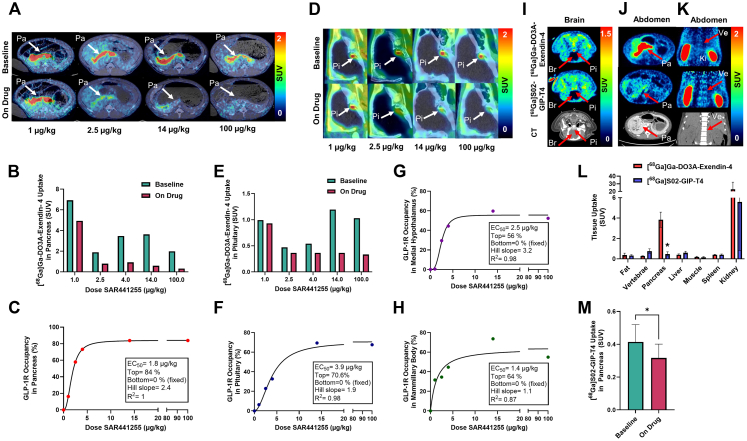
A) Representative PET/CT images of [^68^Ga] Ga-DO3A-Exendin-4 uptake in the pancreas (Pa) at before (Baseline) and after (On Drug) the injection of varying doses of SAR441255. (B) Quantification of individual SUV values in the pancreas at baseline and following SAR441255 administration at different doses. (C) GLP-1R occupancy in pancreas plotted against log_10_-transformed SAR441255 doses. (D) PET/CT images of [^68^Ga]Ga-DO3A-Exendin-4 uptake in the pituitary gland (Pi) at before (Baseline) and after (On Drug) the injection of varying doses of SAR441255. (E) The SUV variation in the pituitary between the first and second scans at different SAR441255 doses. (F) GLP-1R occupancy in the pituitary gland plotted against SAR441255 doses. (G) GLP-1R occupancy in medial hypothalamus and (H) mammillary body plotted against SAR441255 doses. (I–K) Direct comparison of *in vivo* biodistribution and binding of [^68^Ga]Ga-Exendin-4 and [^68^Ga]S02-GIP-T4 in pig. Representative images are shown of the brain (Br = inside brain), (I; Pi = pituitary) and abdomen (J, transaxial projection; Pa = pancreas) (K, coronal projection; Ki = kidney, Ve = Vertebrae, Sc = Subcutaneous fat). (L) A quantitative comparison of the uptake of [^68^Ga]S02-GIP-T4 and [^68^Ga]Ga-DO3A-Exendin-4 in different tissues (∗*p* < 0.05). (M) Quantification of [^68^Ga]S02-GIP-T4 uptake in the pancreas before and after pre-treatment with 100 μg/kg SAR44125 (∗*p* < 0.05).

At baseline scans, [^68^Ga]Ga-DO3A-Exendin-4 demonstrated robust binding to tissues such as the pancreas, although individual variation in uptake was noted (Fig. 2A–C). The pronounced individual variability in baseline pancreatic SUVs (∼2–7) aligns with prior observations in GLP-1R PET imaging of individuals with type 2 diabetes,^23^ in which pancreatic uptake of [^68^Ga]Ga-DO3A-Exendin-4 at the baseline ranged from SUV of 2 to over an SUV of 6.

This variability likely arises from physiological heterogeneity in pancreatic GLP1-R expression and tissue composition. Similarly, the pituitary gland/neurohypophysis region exhibited pronounced and clearly detectable binding (Fig. 2D and E), consistent with previous reports indicating high GLP-1R expression in this region.^3^ This finding aligns well with established GLP-1R localisation and expression patterns within the body.^3^

##### SAR441255

The uptake of [^68^Ga]Ga-DO3A-Exendin-4 by the pancreas was reduced by 16% when pre-injected with 1 μg/kg of SAR441255, by 56% when pre-treated with 2.5 μg/kg, and by 73% when pre-treated with 4 μg/kg of SAR441255 (Fig. 2A–C). The maximum receptor occupancy was achieved at 14 μg/kg SAR441255, reducing the GLP-1R receptor availability in the pancreas by 83%, which remained the same even after pre-injecting with an even higher dose of SAR441255 (100 μg/kg) (Fig. 2C). The estimated EC_50_ in the pancreas was 1.8 μg/kg.

The uptake of [^68^Ga]Ga-DO3A-Exendin-4 in the pituitary was reduced by 77% when pre-injected with 14 μg/kg of SAR441255 (Fig. 2D and E). Furthermore, the data demonstrate a clear correlation between the GLP-1 receptor occupancy of SAR441255 in the pituitary and the injected dose of SAR441255 (Fig. 2F). Although the absolute binding in the medial hypothalamic area was low, it was reduced dose-dependently by SAR441255, indicating target engagement of up to 60% at the GLP-1R in this region, which includes the arcuate nucleus and the periventricular nucleus (Fig. 2G). A similar dose-dependent occupancy of SAR441255 was seen in the mammillary body, also known to express GLP-1R (Fig. 2H). Most other brain regions with BBB demonstrated negligible uptake of [^68^Ga]Ga-DO3A-Exendin-4 at baseline.

The estimated EC_50_ in the pituitary and medial hypothalamic area was 3.9 μg/kg and 2.5 μg/kg, respectively, which was higher than in the pancreas.

##### Tirzepatide

**Fig. 3:**
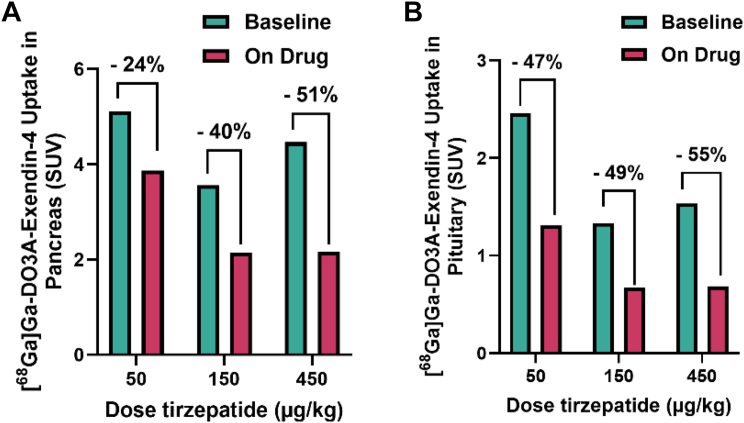
Reduction in [^68^Ga]Ga-DO3A-Exendin-4 uptake in (A) pancreas and (B) pituitary following administration of different doses of tirzepatide.

The occupancy at GLP-1R of tirzepatide in both the pancreas and pituitary did not exceed 60%, even at the highest doses (450 μg/kg) (Fig. 3A and B). Additionally, the occupancy was not dose-dependent, likely indicating a limitation of the current study design, where occupancy of tirzepatide was evaluated much earlier than at T_max_. In general, SAR441255 demonstrated higher occupancy in most GLP-1R expressing compared to tirzepatide, at comparable time points after drug administration.

##### Biodistribution comparison between [^68^Ga]Ga-DO3A-Exendin-4 and [^68^Ga]S02-GIP-T4

A direct comparison of [^68^Ga]Ga-DO3A-Exendin-4 and [^68^Ga]S02-GIP-T4 tracers demonstrated that [^68^Ga]S02-GIP-T4 exhibited 7–10 times weaker binding in the pancreas compared to [^68^Ga] Ga-DO3A-Exendin-4 and 3–4 times weaker uptake in the pituitary gland at 60 min post-tracer injection (Fig. 2I–M). Additionally, [^68^Ga]S02-GIP-T4 showed somewhat increased binding in vertebrae and bone marrow compared to [^68^Ga]Ga-DO3A-Exendin-4 (Fig. 2K).

Furthermore, [^68^Ga]S02-GIP-T4 exhibited higher hepatic retention than [^68^Ga]Ga-DO3A-Exendin-4, likely due to increased hepatic metabolism rather than binding, as no consistent reduction in hepatic signal was observed following study drug administration intended to block GIPR (Fig. 2L).

#### Occupancy at the GIPR

The GIPR occupancy for SAR441255 was assessed by measuring the reduction in [^68^Ga]S02-GIP-T4 uptake following administration of the study drugs, relative to the tracer's baseline uptake.

##### SAR441255

[^68^Ga]S02-GIP-T4 revealed measurable occupancy at the GIPR after administration of SAR441255. Following administration of SAR441255, pancreatic signal intensity significantly decreased by 23 ± 8.5% (*p* < 0.05, paired t-test) relative to baseline (Fig. 2M). A similar reduction of 24% was observed after infusion of GIP(1–42). In contrast to the results obtained with [^68^Ga]Ga-DO3A-Exendin-4, no significant changes in signal intensity were detected in the brain or other organs after administration of the study drugs, which could be attributed to low target density in central regions, limited tracer penetration across the BBB, and a reduced signal-to-noise ratio, together limiting the ability to detect receptor occupancy *in vivo*.

## Discussion

There is a lack of specific and non-invasive technologies and biomarkers to study the target engagement of the new class of unimolecular dual or triple incretin mimetics. Improved understanding of the mechanism of action of these drugs in both the periphery and in the CNS is necessary for further progression in harnessing their potential in the treatment of both metabolic disease and obesity.

In this study, we demonstrate that drug occupancy at GLP-1R can be evaluated both in the pancreas and, for the first time, in brain regions with limited BBB protection, specifically in the appetite centre of the medial hypothalamus, using [^68^Ga]Ga-DO3A-Exendin-4. In contrast, drug occupancy at GIPR could only be evaluated in the pancreas, as no significant changes in [^68^Ga]S02-GIP-T4 brain signal were observed following administration of the study drugs.

[^68^Ga]Ga-DO3A-Exendin-4 demonstrated a strong potential for assessing GLP-1R drug occupancy in the pig model *in vivo*. Both SAR441255 and tirzepatide demonstrated competition with [^68^Ga]Ga-DO3A-Exendin-4 in an *in vitro* autoradiography binding assay, indicating that they bind to the same binding site at the GLP-1R. Thus, [^68^Ga]Ga-DO3A-Exendin-4 can be used to investigate target engagement of these drugs *in vivo* by PET.

SAR441255 demonstrated a strong correlation between the administered dose and the GLP-1R occupancy in relevant tissues, e.g., pancreas, as measured by a decrease in [^68^Ga]Ga-DO3A-Exendin-4 binding after the administration of the study drug. The estimated EC_50_ for SAR441255 in the pancreas was 1.8 μg/kg, indicating the subcutaneous dose required to reach a 50% receptor binding in this tissue. To our knowledge, there is no other technology available to quantify direct receptor occupancy in individual tissues non-invasively.

By contrast, the receptor occupancy of tirzepatide did not increase dose-dependently when the dose was doubled or tripled, even at doses far above the approved dose per kilogram for humans (Fig. 3). This can likely be explained by the lower GLP-1R affinity of tirzepatide, which instead is shifted towards higher affinity to the GIPR.^26^ *In vitro*, tirzepatide demonstrated a fivefold lower affinity to GLP-1R than the native GLP-1 hormone, while maintaining the same affinity to GIPR as endogenous GIP(1–42).^26^ On the contrary, SAR441255 exhibits comparable affinities to GLP-1R, GIPR, and GCGR as observed with the endogenous ligands.^13^ Another contributing factor to the weaker receptor occupancy of tirzepatide compared to SAR441255 observed at these conditions here can be explained by its pharmacokinetic (PK) properties. The maximal plasma concentration (T_max_) following subcutaneous administration of tirzepatide ranged from 8 to 72 h, whereas SAR441255 reaches T_max_ approximately 3 h post-injection.^13^^,^^27^ Thus, this particular model and study design are optimal for the evaluation of SAR441255 and compounds with similar PK properties. On the other hand, it is suboptimal for evaluating compounds with longer circulatory half-life driven by increased albumin binding via the carboxylic acid containing fatty acid side chain, such as tirzepatide. However, this is the disadvantage of the current pig large animal model and study design, where the animal cannot be kept under anaesthesia overnight. In humans, it is straightforward to design a study where the follow-up scan is performed days or weeks after study drug administration, allowing tirzepatide to reach its T_max_ before rescanning for drug occupancy.

The PET/CT scans confirmed SAR441255 and tirzepatide target to pancreatic GLP-1R *in vivo*. Additionally, the scans demonstrated GLP-1R occupancy in the pituitary gland (also known as the neurohypophysis) by both SAR441255 and tirzepatide. The pituitary is a CVO, which is outside the BBB, and thus even large and charged peptides such as [^68^Ga]Ga-DO3A-Exendin-4 can easily access receptors in this tissue. However, SAR441255 exhibited an overall higher occupancy at the GLP-1R compared to tirzepatide, even when compared with the higher doses administered for the latter. The GLP-1R occupancy in the pituitary gland for both SAR441255 and tirzepatide was strong and in the case of SAR441255 increased dose-dependently with the increased dose of the injection. In an earlier study evaluating the potential targeting of Exendin 4-PET in human, it was demonstrated that the pituitary exhibited a very strong expression of GLP-1R, which is in line with the clear visualisation of this tissue seen here.^28^ Furthermore, the hypothalamic-pituitary axis (HPA) is a complex neuroendocrine system that plays a crucial role in regulating various physiological processes in the body, including metabolism.^29^ GLP-1 receptors have been localised in previously mentioned brain regions and are important for the regulation of satiety.^3^

Importantly, SAR441255 GLP-1R occupancy was also evident in some areas with partial protection of the BBB, particularly in the medial hypothalamic area and the mammillary body (Fig. 2G and H). Even though it has been proposed that Exendin-4-based PET tracers cannot readily penetrate the BBB,^30^ the tracer could potentially get access to the hypothalamus through receptor-mediated transcytosis by hypothalamic tanycyte cells. These highly specialised ependymoglial cells function as gatekeepers by facilitating signal transport between the brain parenchyma and the cerebrospinal fluid (CSF).^31^ Considering that the tanycytic route of entry has been observed for certain peptide-based GLP-1R agonists, such as liraglutide, these cells may also function as an entry point for the Exendin-4 tracer into brain regions that are usually protected by the BBB.^32^ GLP-1R expression in the mammillary body was found throughout the supramammillary nucleus (SuM), and importantly, directly on neurons connecting to the lateral hypothalamus LH, suggesting that GLP-1R activation in the SuM might alter communication between these two brain regions in rats.^33^ Activation of SuM GLP-1R suppressed chow consumption in rats and demonstrated a reduction in motivated behaviour for sucrose.^34^ Furthermore, the stimulation of GLP-1R in the ventral tegmental area is responsible for feeding-oriented behaviour and has a suppressive effect on food reward/motivation.^34^ Overall, GLP-1R activation in the CNS in rats and mice has been shown to be effective in reducing the consumption of rewarding substances other than food, such as cocaine and alcohol.^35^

The GLP-1R occupancy of SAR441255 in CVOs and brain regions, including medial hypothalamus (incorporating arcuate nucleus and periventricular nucleus), was dose-dependent. The presence of GLP-1R has been reported in various brain areas, including circumventricular organs (CVOs) where GLP-1R are highly abundant.^36^ Unlike the BBB that acts as a shield against entry of polar and large molecules, the CVOs are surrounded by an extensive network of permeable blood vessels, facilitating the exchange of molecules and neurons with the bloodstream.^37^

Interestingly, the dose-occupancy relationship for SAR441255 was shifted to the right on the x-axis compared to that for the pancreas, i.e., the estimated EC_50_ in pituitary (3.9 μg/kg) and medial hypothalamus (2.5 μg/kg) was higher than in the pancreas (1.8 μg/kg). This indicates that higher doses of a GLP-1R agonist are required to achieve receptor occupancy in CVOs and brain regions. While this observation requires further investigation, it is reasonable given the reduced drug access to brain receptors due to partial BBB protection. Additionally, these findings indicate that higher doses of SAR441255 or similar GLP-1R mono- or multi-agonists may be required to elicit centrally mediated effects on appetite and weight control than the dose needed for the incretin effect in the pancreas.

On the other hand, assessing GIPR drug occupancy in the pig model using [^68^Ga]S02-GIP-T4 proved to be more challenging. While [^68^Ga]S02-GIP-T4 tracer exhibited good specificity for GIPR *in vitro*, its *in vivo* binding was weak, even in organs with known expression of GIPR, such as the pancreas. The baseline SUV signal in the pancreas was already low, suggesting limited tracer retention. Pre-treatment with SAR441255, intended to assess GIPR occupancy, did not lead to a strong reduction in tracer uptake within the pancreas. Although a consistently reduced signal in the follow-up scan, post drug administration, was observed (23 ± 8.5%), it remains unclear whether this reflects actual GIPR occupancy or other physiological factors.

This contrasts with previously reported results, where tracer [^68^Ga]S02-GIP-T4 demonstrated higher *in vivo* binding in the pancreas in non-human primate (NHP) models, with baseline pancreatic tracer uptake reaching an SUV of ∼1.2 at 60–90 min post-administration.^16^ However, the single-cell transcriptome data suggest that porcine pancreatic GIPR expression is significantly lower than that in humans (Supplementary Figure S1). This reduced receptor density presumably explains the weaker [^68^Ga]S02-GIP-T4 binding and diminished pancreatic PET signal observed in the current study. Also, a minor tracer-induced saturation cannot be entirely excluded. Nevertheless, the injected peptide mass (∼0.1 μg/kg) corresponds to subnanomolar systemic concentrations and is therefore unlikely to result in meaningful receptor saturation. Additionally, [^68^Ga]S02-GIP-T4 exhibited visible retention in the vertebrae, particularly in comparison to [^68^Ga]Ga-DO3A-Exendin-4, which showed no detectable presence in this region. This finding aligns with the physiological distribution of GIPR in bones.^3^^,^^38^

One of the advancements of GLP-1R/GIPR co-activation is potentially the reduced nausea, food aversion, and emesis associated with GLP-1R-based treatment, in addition to an even more increased reduction in appetite and calorie intake.^26^^,^^39^ These effects may be associated with central activation of GLP-1 receptors by these drugs, and it has been proposed that the concurrent activation of central GIPR by tirzepatide exerts an anti-emetic effect, thereby improving tolerability to GLP-1 receptor–mediated side effects. Notably, central administration of endogenous GIP and GLP-1 reduced food intake in mice.^40^ However, there is still a lack of knowledge regarding incretin agonists' target engagement in the CNS when administered peripherally.

Here, we demonstrate that dose-dependent drug occupancy of incretin mimetics at GLP-1R can be quantified not only peripherally, but also centrally in CVOs and brain regions associated with appetite control. However, tirzepatide did not show detectable occupancy in appetite-regulating brain regions, likely due to the experimental design not aligning with its T_max_. In contrast, single-dose GIPR occupancy with SAR441255 was detectable only in the pancreas and showed no clear evidence of central engagement, further highlighting the limitation of GIPR targeting under the current animal model.

Importantly, this non-invasive PET technology can readily be translated to clinical studies on target engagement, given that [^68^Ga]Ga-DO3A-Exendin-4 is available in Good Manufacturing Practice (GMP) quality at several sites, and can be administered repeatedly without exceeding clinical radiation dosing safety levels.^17^^,^^41^

### Limitations of the study

A key limitation of the porcine animal model used in this study is that GIPR expression in pigs is substantially lower than in humans, as confirmed by single-cell transcriptomic data. This reduced receptor density limits the suitability of the porcine model for assessing GIPR drug occupancy and may restrict the translational relevance of GIPR-related findings. Additionally, the relatively small sample size reduces the statistical power of the study. Furthermore, the inability to detect GIPR occupancy in the brain, likely due to low central receptor expression or limited tracer access, constrains the conclusions about central GIPR engagement. Finally, the study design, constrained by a restricted time window, did not allow for extended protocols or delayed scans. This is particularly relevant for the long-acting compound tirzepatide, where the time to reach maximal plasma concentration, critical for achieving peak receptor occupancy, may exceed the feasible imaging window under the applied study conditions.

### Conclusions and future directions

Our findings demonstrate that PET imaging technology can be used for non-invasive, *in vivo* assessment of dose-dependent GLP-1R occupancy by incretin mimetics in both peripheral and central tissues, and potentially of GIPR occupancy, primarily in the pancreas. While the results support the translational potential of this approach, further validation across tracers, species, and different dosing conditions is needed to confirm the robustness and generalisability of the method. Future studies should aim to enhance sensitivity for central targets, explore delayed or longitudinal imaging protocols for long-acting compounds, and better define the receptor-specific contributions to the pharmacological effects of dual and triple agonists. Moreover, the use of physiologically relevant models, such as non-human primates with receptor expression patterns similar to those in humans, will be crucial for enhancing translational accuracy and validating occupancy readouts for clinical applications.

## Contributors

A.K. wrote the manuscript and performed, analysed, and interpreted the studies. I.V. edited the manuscript, conceived, planned, and interpreted the studies, and performed the radiochemistry. M.X. edited the manuscript and interpreted the studies, M.B. and M.W. edited the manuscript, conceived the studies, designed and synthesised the PET precursor and drug compounds. O.E. conceived, planned, and interpreted the studies, was responsible for funding acquisition, and wrote the manuscript. A.K. and O.E. accessed and verified the underlying data. All co-authors read and approved the final version of the manuscript.

## Data sharing statement

All the data in the main text are available within the article and/or the Supplementary Materials. For further information, contact the corresponding author at olof.eriksson@ilk.uu.se.

## Declaration of interests

O.E. is an employee, co-founder and minority shareholder of Antaros Tracer AB. M.B. and M.W are the inventors on the patent of SAR441255 and GIP PET tracer, has shares and stocks options in Sanofi. MB is an employee of Sanofi and discloses that Sanofi provided SAR441255 and a precursor to the GIP PET tracer used in the study.

M.W. is an employee of Dewpoint Therapeutics as well as a cofounder and minority shareholder of Antaros Tracer. Otherwise, the authors declare no competing interests.
