# Supplementary material for: Positron emission tomography to assess drug occupancy at peripheral and central incretin receptors
Source: eBioMedicine. 2025 Nov 21;122:106033. doi: 10.1016/j.ebiom.2025.106033 (PMC12682123; doi:10.1016/j.ebiom.2025.106033)
Supplement: Supplementary Tables [file mmc1.docx]

**Table S1.** Dynamic time–activity data of the pituitary obtained from pigs (n = 5) treated with different doses of SAR441255 and imaged with the [^68^Ga]Ga-DO3A-Exendin-4 tracer.

| **Time (min)** | **SAR441255**  **1 (μg /kg)** | | **SAR441255**  **2.5 (μg /kg)** | | **SAR441255**  **4 (μg /kg)** | | **SAR441255**  **14 (μg /kg)** | | **SAR441255**  **100 (μg /kg)** | |
| --- | --- | --- | --- | --- | --- | --- | --- | --- | --- | --- |
|  | **Baseline_SUV_** | **On Drug_SUV_** | **Baseline_SUV_** | **On Drug_SUV_** | **Baseline_SUV_** | **On Drug_SUV_** | **Baseline_SUV_** | **On Drug_SUV_** | **Baseline_SUV_** | **On Drug_SUV_** |
| 0.25 | 4.30 | 0.28 | 0.09 | 0.69 | 0.74 | 0.03 | 0.00 | 0.97 | 1.13 | 1.85 |
| 1 | 3.64 | 3.39 | 3.79 | 1.31 | 1.47 | 2.75 | 0.76 | 1.84 | 1.00 | 3.92 |
| 2 | 6.28 | 0.52 | 0.27 | 1.90 | 1.78 | 2.13 | 1.78 | 1.47 | 0.89 | 1.66 |
| 5 | 2.13 | 3.07 | 0.44 | 1.53 | 1.05 | 2.27 | 1.23 | 0.72 | 1.72 | 0.89 |
| 10 | 1.45 | 1.25 | 0.76 | 0.54 | 1.57 | 1.38 | 1.20 | 1.15 | 1.17 | 0.68 |
| 20 | 1.14 | 1.48 | 0.55 | 0.68 | 1.20 | 0.91 | 1.41 | 0.70 | 1.12 | 0.60 |
| 30 | 0.99 | 1.36 | 0.56 | 0.46 | 0.94 | 0.84 | 0.95 | 0.45 | 0.85 | 0.44 |
| 45 | 1.02 | 1.04 | 0.58 | 0.52 | 0.73 | 0.58 | 1.33 | 0.33 | 0.97 | 0.40 |
| 55 | 0.99 | 0.93 | 0.47 | 0.36 | 0.68 | 0.50 | 1.15 | 0.37 | 1.03 | 0.33 |

**Table S2.** Dynamic time–activity data of the hypothalamus obtained from pigs (n = 5) treated with different doses of SAR441255 and imaged with the [^68^Ga]Ga-DO3A-Exendin-4 tracer.

| **Time (min)** | **SAR441255**  **1 (μg /kg)** | | **SAR441255**  **2.5 (μg /kg)** | | **SAR441255**  **4 (μg /kg)** | | **SAR441255**  **14 (μg /kg)** | | **SAR441255**  **100 (μg /kg)** | |
| --- | --- | --- | --- | --- | --- | --- | --- | --- | --- | --- |
|  | **Baseline_SUV_** | **On Drug_SUV_** | **Baseline_SUV_** | **On Drug_SUV_** | **Baseline_SUV_** | **On Drug_SUV_** | **Baseline_SUV_** | **On Drug_SUV_** | **Baseline_SUV_** | **On Drug_SUV_** |
| 0.25 | 0.33 | 0.05 | 0.04 | 0.95 | 0.00 | 0.00 | 0.00 | 0.26 | 0.71 | 0.23 |
| 1 | 1.31 | 0.81 | 0.36 | 0.59 | 0.40 | 0.67 | 0.10 | 0.30 | 0.08 | 0.16 |
| 2 | 1.20 | 0.64 | 0.74 | 0.95 | 0.31 | 0.85 | 0.35 | 0.39 | 0.20 | 0.43 |
| 5 | 0.67 | 0.44 | 0.75 | 0.47 | 0.58 | 0.51 | 0.29 | 0.32 | 0.37 | 0.20 |
| 10 | 0.49 | 0.29 | 0.36 | 0.27 | 0.35 | 0.49 | 0.19 | 0.35 | 0.17 | 0.22 |
| 20 | 0.36 | 0.48 | 0.32 | 0.31 | 0.38 | 0.36 | 0.28 | 0.17 | 0.22 | 0.13 |
| 30 | 0.37 | 0.29 | 0.25 | 0.24 | 0.44 | 0.30 | 0.21 | 0.13 | 0.22 | 0.07 |
| 45 | 0.41 | 0.32 | 0.24 | 0.21 | 0.34 | 0.21 | 0.20 | 0.10 | 0.28 | 0.09 |
| 55 | 0.41 | 0.30 | 0.29 | 0.21 | 0.37 | 0.21 | 0.21 | 0.09 | 0.17 | 0.08 |

**Table S3.** Dynamic time–activity data of the mammillary body obtained from pigs (n = 5) treated with different doses of SAR441255 and imaged with the [^68^Ga]Ga-DO3A-Exendin-4 tracer.

| **Time (min)** | **SAR441255**  **1 (μg /kg)** | | **SAR441255**  **2.5 (μg /kg)** | | **SAR441255**  **4 (μg /kg)** | | **SAR441255**  **14 (μg /kg)** | | **SAR441255**  **100 (μg /kg)** | |
| --- | --- | --- | --- | --- | --- | --- | --- | --- | --- | --- |
|  | **Baseline_SUV_** | **On Drug_SUV_** | **Baseline_SUV_** | **On Drug_SUV_** | **Baseline_SUV_** | **On Drug_SUV_** | **Baseline_SUV_** | **On Drug_SUV_** | **Baseline_SUV_** | **On Drug_SUV_** |
| 0.25 | 0.89 | 0.15 | 0.06 | 1.07 | 0.00 | 0.00 | 0.02 | 0.16 | 1.25 | 1.05 |
| 1 | 1.00 | 1.64 | 0.87 | 0.33 | 0.72 | 0.52 | 0.55 | 1.11 | 0.14 | 1.28 |
| 2 | 1.50 | 0.28 | 0.20 | 1.68 | 0.52 | 0.88 | 0.49 | 1.10 | 0.63 | 0.92 |
| 5 | 1.18 | 0.39 | 0.18 | 0.86 | 0.48 | 0.48 | 1.13 | 0.42 | 0.87 | 0.32 |
| 10 | 0.50 | 0.39 | 0.33 | 0.34 | 0.58 | 0.64 | 0.56 | 0.57 | 0.30 | 0.22 |
| 20 | 0.48 | 0.57 | 0.30 | 0.40 | 0.42 | 0.39 | 0.55 | 0.30 | 0.24 | 0.19 |
| 30 | 0.42 | 0.32 | 0.27 | 0.30 | 0.38 | 0.31 | 0.58 | 0.32 | 0.23 | 0.22 |
| 45 | 0.52 | 0.48 | 0.30 | 0.24 | 0.41 | 0.32 | 0.58 | 0.14 | 0.35 | 0.22 |
| 55 | 0.63 | 0.43 | 0.32 | 0.21 | 0.42 | 0.23 | 0.58 | 0.15 | 0.29 | 0.13 |

**Table S4**. Dynamic time–activity data of the pituitary obtained from pigs (n = 3) treated with different doses of tirzepatide and imaged with the [^68^Ga]Ga-DO3A-Exendin-4 tracer.

| **Time (min)** | **tirzepatide**  **50 (μg /kg)** | | **tirzepatide**  **150 (μg /kg)** | | **tirzepatide**  **450 (μg /kg)** | |
| --- | --- | --- | --- | --- | --- | --- |
|  | **Baseline_SUV_** | **On Drug_SUV_** | **Baseline_SUV_** | **On Drug_SUV_** | **Baseline_SUV_** | **On Drug_SUV_** |
| 0.25 | 0.40 | 3.24 | 0.00 | 0.00 | 0.22 | 0.24 |
| 1 | 0.79 | 0.97 | 1.20 | 0.90 | 0.80 | 1.61 |
| 2 | 1.74 | 0.68 | 1.58 | 1.39 | 1.14 | 2.33 |
| 5 | 1.71 | 1.76 | 1.82 | 1.57 | 0.88 | 2.16 |
| 10 | 1.81 | 1.51 | 1.36 | 1.21 | 1.25 | 1.44 |
| 20 | 1.45 | 1.47 | 1.34 | 1.15 | 1.19 | 1.36 |
| 30 | 1.59 | 1.53 | 1.18 | 1.03 | 1.21 | 1.24 |
| 45 | 1.51 | 1.63 | 1.11 | 0.95 | 0.98 | 0.87 |
| 55 | 1.67 | 1.65 | 1.12 | 0.91 | 0.99 | 1.07 |

**Table S5**. Dynamic time–activity data of the pancreas obtained from pigs (n = 2) treated with the same dose of SAR441255 and imaged with the [^68^Ga]S02-GIP-T4 tracer.

| **Time (min)** | **Animal 1** | | **Animal 2** | |
| --- | --- | --- | --- | --- |
|  | **SAR441255**  **100 (μg /kg)** | | **SAR441255**  **100 (μg /kg)** | |
|  | **Baseline_SUV_** | **On Drug_SUV_** | **Baseline_SUV_** | **On Drug_SUV_** |
| 0.25 | 0.91 | 3.03 | 1.24 | 4.11 |
| 1 | 3.02 | 1.64 | 1.66 | 1.83 |
| 2 | 1.10 | 1.38 | 1.25 | 1.60 |
| 5 | 0.93 | 1.36 | 1.24 | 0.79 |
| 10 | 0.80 | 0.69 | 0.79 | 1.06 |
| 20 | 0.56 | 0.64 | 0.69 | 0.59 |
| 30 | 0.61 | 0.43 | 0.65 | 0.52 |
| 45 | 0.56 | 0.24 | 0.48 | 0.38 |
| 55 | 0.52 | 0.32 | 0.42 | 0.36 |
