# Supplementary material for: Positron emission tomography to assess drug occupancy at peripheral and central incretin receptors
Source: eBioMedicine. 2025 Nov 21;122:106033. doi: 10.1016/j.ebiom.2025.106033 (PMC12682123; doi:10.1016/j.ebiom.2025.106033)
Supplement: Supplementary Figures [file mmc2.docx]

**Supplementary Material**


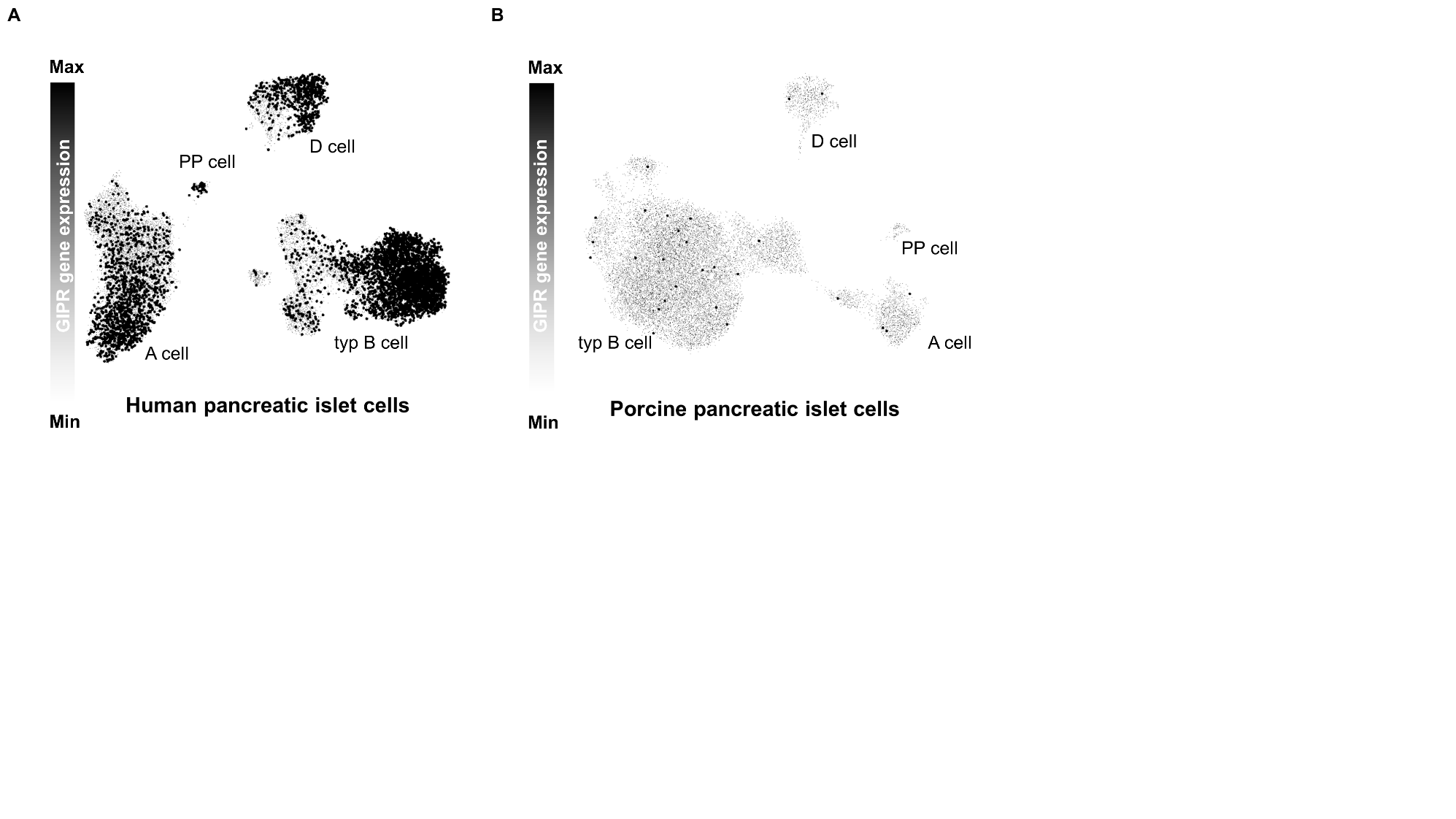


**Figure S1**. Uniform Manifold Approximation and Projection (UMAP) visualization of single-cell transcriptomes from (A) human and (B) pig pancreatic islet cells.

UMAP visualizations were generated using CELLxGENE (Chan Zuckerberg Initiative).


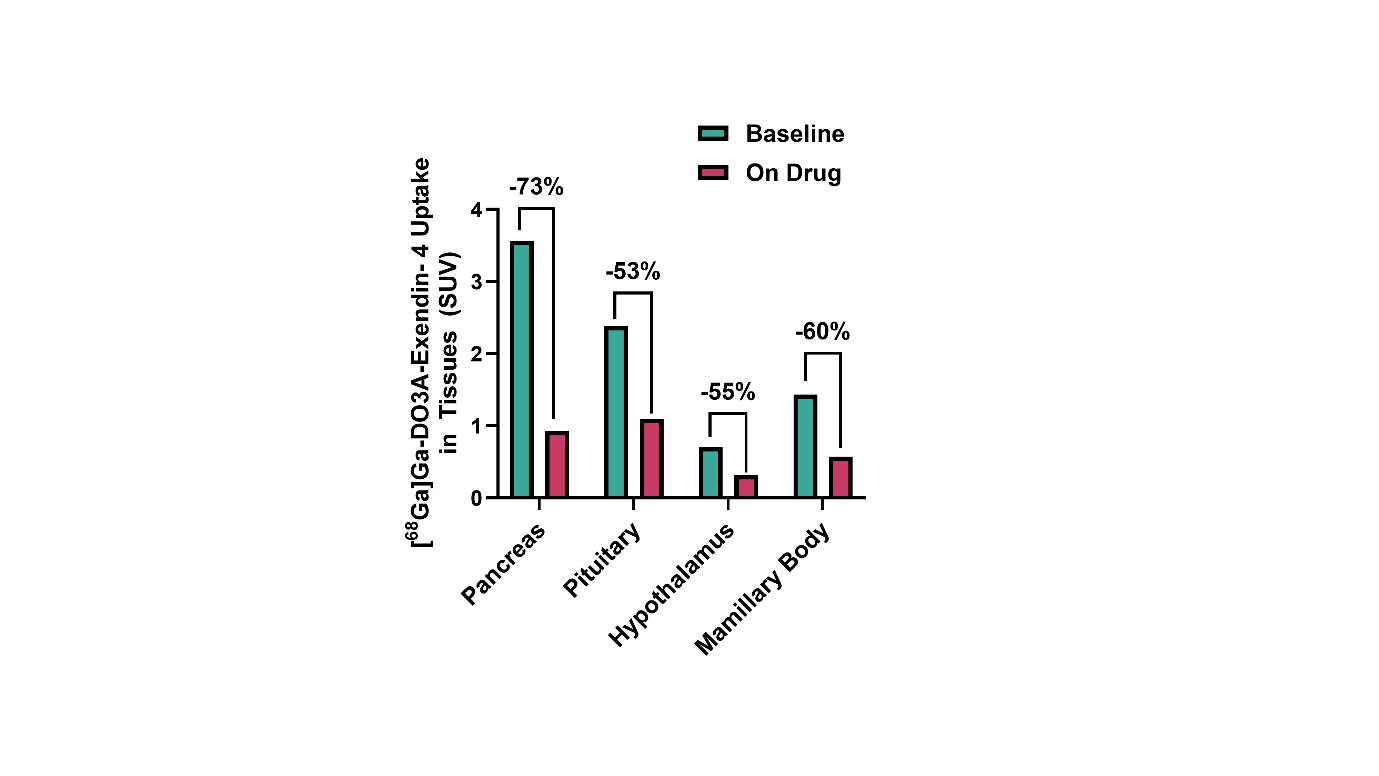


**Figure S2**. Positive control: Reduction in [^68^Ga] Ga-DO3A-Exendin-4 uptake in various organs following administration of DO3A-VS-Exendin-4.
